# Formulation of Mesoporous Silica Nanoparticles for Controlled Release of Antimicrobials for Stone Preventive Conservation

**DOI:** 10.3389/fchem.2020.00699

**Published:** 2020-08-21

**Authors:** Alessandro Presentato, Francesco Armetta, Alberto Spinella, Delia Francesca Chillura Martino, Rosa Alduina, Maria Luisa Saladino

**Affiliations:** ^1^Department of Biological, Chemical, and Pharmaceutical Sciences and Technology (STEBICEF), University of Palermo, Palermo, Italy; ^2^Advanced Technologies Network (ATeN) Center, University of Palermo, Palermo, Italy

**Keywords:** mesoporous silica nanoparticles, controlled release systems, condensation in emulsion, biocides, biodeterioration, stone conservation, cultural heritage

## Abstract

The biotic deterioration of artifacts of archaeological and artistic interest mostly relies on the action of microorganisms capable of thriving under the most disparate environmental conditions. Thus, to attenuate biodeterioration phenomena, biocides can be used by the restorers to prevent or slow down the microbial growth. However, several factors such as biocide half-life, its wash-out because of environmental conditions, and its limited time of action make necessary its application repeatedly, leading to negative economic implications. Sound and successful treatments are represented by controlled release systems (CRSs) based on porous materials. Here, we report on the design and development of a CRS system based on mesoporous silica nanoparticles (MSNs), as a carrier, and loaded with a biocide. MSNs, with a diameter of 55 nm and cylindrical pores of ca. 3–8 nm arranged as parallel arrays concerning the NP diameter, and with 422 m^2^/g of specific surface area were synthesized by the sol-gel method assisted by oil in water emulsion. Biocide loading and release were carried out in water and monitored by UV-Vis Spectroscopy; in addition, microbiological assay was performed using as control the MCM-41 mesoporous silica loaded with the same biocide. The role of specific supramolecular interaction in regulating the release is discussed. Further, we demonstrated that this innovative formulation was useful in inhibiting the *in vitro* growth of *Kocuria rhizophila*, an environmental Gram-positive bacterial strain. Besides, the CRS here prepared reduced the bacterial biomass contaminating a real case study (i.e., stone derived from the Santa Margherita cave located in Sicily, Italy), after several months of treatment thus opening for innovative treatments of deteriorated stone artifacts.

## Introduction

Mesoporous silica nanoparticles (MSNs) are one of the most interesting recent findings in the field of materials chemistry (Farjadian et al., 2019). Thanks to the high percentage of hydroxyl groups in the inner walls of the pores and the high thermal and chemical stability and biocompatibility, MSNs have been used as nanoreactors for catalytic applications (Zhu et al., 2019) and as controlled release systems (CRSs) in the biological medicine (Slowing et al., 2008; Niu et al., 2014; Niculescu, 2020). Furthermore, MSNs have a high adsorption capacity guaranteed by the high value of pore volume and surface area, being their surface properties easily modifiable. Other interesting features of MSNs are their inert and biodegradable nature (Barbé et al., 2004; Lu et al., 2007; Trewyn et al., 2008; Lin and Haynes, 2009). As far as the drug delivery issue is concerned, MSNs are considered very promising because of their capacity in loading larger amounts of drugs as compared to conventional mesoporous materials (Du et al., 2013; Ma et al., 2013; Niu et al., 2014; Lu et al., 2015; Bernardos et al., 2019).

The development of alternative strategies based on either metal or metal-oxide nanoparticles (Russo et al., 2015, 2016; Qi et al., 2017, 2019, 2020; Cusimano et al., 2020; Yaqoob et al., 2020), as well as mesoporous ones (Cavallaro et al., 2018) and natural biocides (Fidanza and Caneva, 2019) has gained momentum mainly when it comes to the prevention of manufacture goods belonging to cultural heritage from chemical- or bio-deterioration phenomena (Artesani et al., 2020; David et al., 2020). Particularly, CRSs based on mesoporous materials have been developed to protect both environment and artifacts of archaeological and artistic interest (Popat et al., 2012; Ruggiero et al., 2019). Recent studies showed that the mesoporous silica can release biocides “on-demand” (Böttcher et al., 1999; Chan et al., 2017; Ruggiero et al., 2018). In this regard, the long-term actions of MSNs on paper (Michaelsen et al., 2013), woods (Liu et al., 2001), and stones (Allsopp et al., 2004; Eyssautier-Chuine et al., 2018) have been already assessed.

The development of CRSs of biocide for the preventive conservation of artifacts derives from the need to control the deteriogenic action of microorganisms on them. Indeed, bacteria and fungi contaminating artifacts can determine the rise of detrimental phenomena of the work of arts themselves, as microorganisms can release a vast array of metabolites (i.e., oxalic, citric, and sulphuric acids), extracellular enzymes, biological pigments, carotenoids, to name a few (Warscheid and Braams, 2000; Urzì, 2004). Thus, the interaction occurring between these biotic compounds and artifacts can lead to physicochemical and aesthetic alterations of the latter, demanding their treatment with expensive procedures. During a restoration work aimed to restrain the action of deteriogenic microorganisms, the restorers apply products whose efficacy would depend on the species to be contained, the artifacts' features, as well as physicochemical properties of the chosen product and its mode of application. Nowadays, most biocides feature high toxicity against both environment and operator, resulting in potentially noxious for human health especially in the case of museum environments. In this context, CRSs are of utmost interest as they could reduce the number of treatments over time concurrently guaranteeing the artifact protection.

With all this in mind, here, a new system consisting of mesoporous silica nanoparticles (MSNs) for the controlled release of biocides, long-term preventing and preserving both archaeological sites and museum environments from biodeterioration phenomena, is proposed. Specifically, the CRS was designed for the preventive conservation of stone material. MSNs were prepared through the method of condensation in an emulsion and characterized by several physicochemical and biological analyses. Preventol RI-80 is a commercial biocide commonly used by restorers constituted by a mixture of quaternary ammonium salts, where the main component is benzyldimethyltridecylazanium chloride. In this study, it was chosen as a biocide, since it is active against a wide variety of fungi, algae, and bacteria contaminating stone materials, plasters, wood, and ceramics. Preventol RI-80 loading on and its release from MSNs were performed in water; further, the efficiency of the best CRS obtained was compared to that of mesoporous silica MCM-41 (Dresler et al., 2017). The validity of this formulation was supported by the application of the best CRS, in terms of the biocide release performance, on a real case study consisting of stone fragments deriving from a cave (named as *Grotta di Santa Margherita*) located in Castellammare del Golfo (Trapani, Italy). The ability of the system to locally kill microorganisms once applied on the stone materials was evaluated over 12 months, evaluating the microbial proliferation every 3 months, strengthening the suitable application of such material for the prevention of work of arts.

## Materials and Methods

### Materials

Tetraethyl ortosilicate (TEOS, *d* = 0.934 g/mL 99%, Aldrich), cetyl-trymethyl ammonium bromide (CTAB 98%, Aldrich), ethanol (*d* = 0.789 g/mL ≥99.8%, Fluka), *n*-heptane (*d* = 0.688 g/mL 99%, Aldrich), ammonium hydroxide (*d* = 0.90 g/mL, 30%, Carlo Erba), and hydrochloric acid (37%, Aldrich) were used without further purification. The synthesis of the MCM-41 was performed as reported elsewhere (Caponetti et al., 2010; Saladino et al., 2011; Lavall et al., 2012). MCM-41 had highly ordered hexagonal structures with toroidal particles of few microns. The specific surface area S_BET_ and the average pore size w_BJH_ were 931 m^2^/g and 2.4 nm, respectively. Preventol RI-80 was supplied by C.T.S. s.r.l. (Altavilla Vicentina (VI), Italy), being no information provided about the purity of the formulation. Aqueous solutions were prepared by weight, using conductivity grade water having 1.5 μS/m of conductivity.

### MSNs Synthesis

MSNs were synthesized following the emulsion-condensation route reported by Cao et al. (2016), which involves the hydrolysis of alkoxysilanes followed by the reaction of polycondensation of the resulting silanol on a template consisting of CTAB surfactant molecules (Ma et al., 2011; Xu et al., 2014; Cao et al., 2016; Farjadian et al., 2019; Zhu et al., 2019). The oil phase (*n*-heptane) determines the size of the nanopores by swelling the micelles and regulating the rate of hydrolysis of alkoxysilanes through the partitioning of the latter among the oil and aqueous phases. Besides, a stabilization of the emulsion either by the surfactant or the formed silica nanoparticles could occur. First, in a Nalgene bottle 70 mL of distilled water, 0.80 mL of ammonium hydroxide, 15 mL of *n*-heptane, 5 mL of ethanol and 0.5 g of CTAB were mixed at room temperature. Once the mixture became homogeneous, 2.6 mL of TEOS were added. The mixture was stirred at room temperature for 4 h. To block the base-catalyzed reaction, 1.0 mL of hydrochloric acid was added to the formed suspension, which was then allowed to stand for 24 h. The obtained product was thus filtered, washed with a mixture 1:1 of water and ethanol to remove CTAB and ammonium chloride formed as byproduct and dried at 60°C for about 72 h, until the weight of the obtained white powder was constant.

### Characterization Techniques

X-ray Diffraction (XRD) patterns were obtained using Philips PW 1050/39 diffractometer in Bragg–Brentano geometry (source Cu Ka, λ = 1.54056 Å, voltage 40 kV, current 30 mA) in the range 2–60°, steps of 0.05° and acquisition time 5 s/step.

The FT-IR spectra were acquired by using FT-IR Bruker Vertex 70 v spectrophotometer with Platinum ATR, with 2 cm^−1^ steps and 60 scans in the acquisition range 4,000–400 cm^−1^. The measure was carried out at 2 hPa. A base line correction of the scattering was made.

NMR spectra were acquired by using a Bruker Advance II 400 spectrometer operating at the frequency of 400.15, 100.62, and 79.49 MHz for the ^1^H, ^13^C, and ^29^Si nuclide, respectively. All samples were placed in 4 mm zirconia rotors equipped with Kel-f caps. ^13^C CPMAS NMR spectrum was acquired with a MAS rotation speed of 7 kHz, at a temperature of 300 K using a 90° pulse on ^1^H of 4.5 μs, a contact time during cross polarization of 2 ms, a delay time of 3 s and 400 scans. ^29^Si CPMAS NMR spectrum was acquired with a MAS rotation speed of 5 kHz, at a temperature of 300 K using a 90° pulse on ^1^H of 4.5 μs, a contact time during cross polarization of 8 ms, a delay time of 5 s and 400 scans. Hartman-Hahn's conditions were optimized by standard samples of adamantane and of tetramethylsilane for ^13^C and ^29^Si nuclei, respectively. The two compounds were also used as external chemical shift reference.

Transmission Electron Microscopy (TEM) investigation was performed by using a JEOL-2100 microscope operating at an accelerating voltage of 200 kV. The powders were dispersed in water and deposited on a copper grid. The observation of the samples was performed after complete evaporation of the solvent. The particle size distribution was determined by linear intercept method based on the TEM micrographs (Dai et al., 2018).

The N_2_ adsorption and desorption isotherms were recorded at 77 K using a Quantachrome Nova 2200 Multi-Station High Speed Gas Sorption Analyzer after degassing of the samples for 24 h at 23°C in the degas station. Adsorbed nitrogen volumes were normalized to the standard temperature and pressure. The specific surface area (S_BET_) was calculated according to the standard BET method in the relative absorption pressure (P/P_0_) range from 0.045 to 0.250 (Brunauer et al., 1938). The total pore volume (V_t_) was obtained from the nitrogen amount adsorbed in correspondence of P/P_0_ equal to 0.99. The cylinder diameter size (w_BJH_) was calculated by the BJH method (Kruk et al., 1999).

UV–vis spectra were recorded in the range 200–500 nm using a double beam Beckman DU-800 spectrophotometer with a resolution of 1.0 nm. To avoid the effect of instrumental errors and of particle diffusion, the value of absorbance at 500 nm was subtracted to each spectrum. A typical spectrum of the Preventol RI-80 is reported in Figure S1.

### Biological Test

The efficiency of the CRS was evaluated by means of disc diffusion antibiotic sensitivity assay, as reported elsewhere (Giardina et al., 2010; Ciabocco et al., 2018), using the Gram-positive bacterial strain *Kocuria rhizophila* ATCC® 9341™ (*K. rhizophila*) since this species is frequently found on works of art and the stone materials (Warscheid, 2003; Randazzo et al., 2015). Briefly, a dense bacterial suspension (~10^7^ cells) was prepared in the Luria Bertani growth medium (hereinafter named as LB and composed of 10 g/L of sodium chloride, 5 g/L of yeast extract, and 10 g/L of tryptone) and spread onto LB-agar-−20% w/v of bacteriological agar—plates. Aqueous suspension aliquots containing different amounts of MSNs and biocides were directly spotted on sterile paper discs (6 mm diameter), which were deposited onto the bacterial overlay present in the LB-agar plate, as previously described (Rubino et al., 2018a). After overnight incubation at 30°C, the diameter of the growth inhibition halos was registered. The antimicrobial activity was calculated as a mean of three replicates and standard deviations were calculated.

The release of biocides from the loaded MSN was followed over time (after 1, 3, 6, 24, and 48 h) by collecting aliquots of the solution in which the CRS was immersed. The aliquots of solution were directly spotted on sterile paper discs and tested as aforementioned to detect antibacterial activity. Twenty microliters of Preventol RI-80 and the dispersions of the two mesoporous materials (0.1_w/v_% MSN and 0.1% _w/v_MCM-41) were tested as controls.

To test the performance of the CRS based on MSNs loaded with Preventol RI-80, total viable bacterial count was measured, as previously described (Piacenza et al., 2018; Poma et al., 2019). Briefly, samples of 1 g of both untreated and treated stones were vigorously vortexed in 10 mL of LB to detach bacteria from the stone material. Serial dilutions of these suspensions were prepared using LB, being 100 μl of each dilution spread onto LB-agar to allow bacterial growth. Plates were incubated at 30°C until colonies appeared. Data are reported as mean (*n* = 3) of the colony forming units (CFU) per g of stone and standard deviations were calculated.

After 1 year, the metagenomic DNA was extracted from these samples using the method reported in Presentato et al. (2020). PCR was carried out with primers and conditions used in Arizza et al. (2019).

### Loading Procedure and Experiments of Release

The biocide loading was performed following the procedure reported elsewhere (Saladino et al., 2016; Dresler et al., 2017; Rubino et al., 2018b). One milligram/millilitre of the mesoporous powder was immersed in the aqueous solutions containing different biocide concentrations (range of the nominal concentration 0.012–0.64 _v/v_%) for 24 h under continuous magnetic stirring in the dark. The values of concentration were chosen based on the restorer's suggestions. The suspension was centrifuged at appropriate RCF for the separation of the powder. Then, the supernatant was carefully removed and the loaded samples dried under vacuum overnight. The loaded MSNs were white and similar to those unloaded. The yield of loading—which was evaluated by UV-vis spectroscopy—was of 100% for all systems. The loaded MSN are called Preventol RI-80 *x*@NP SiO_2_ where *x* is the initial concentration (_v/v_%) of the biocide in which MSNs were immersed. Preventol RI-80 loading within the MSNs was successful, as highlighted by the decreased MSN specific surface area (41 m^2^/g) as compared to that of the unloaded material (422.8 m^2^/g).

The following procedure was carried out to study the release of biocides from the MSN: 20 mg of each biocide-loaded MSN were placed in a closed 3.5 kDa dialysis membrane tube (Spectra/Por 3 Dialysis membrane) and then in a Nalgene-flask filled with 20 mL of water. The flask was kept at room temperature under continuous shaking during all the experiments. The UV-vis spectra were registered on 2.5 mL of solution collected at the scheduled time (1, 3, 6, 24, and 48 h). The release profile was obtained plotting the values of concentration (obtained by Lambert Beer's law) vs. time and evaluating the antibacterial activity by disc diffusion antibiotic sensitivity assays, as described above. Each experiment was performed in triplicate.

## Results and Discussion

### MSNs Characterization

**Figure 1:**
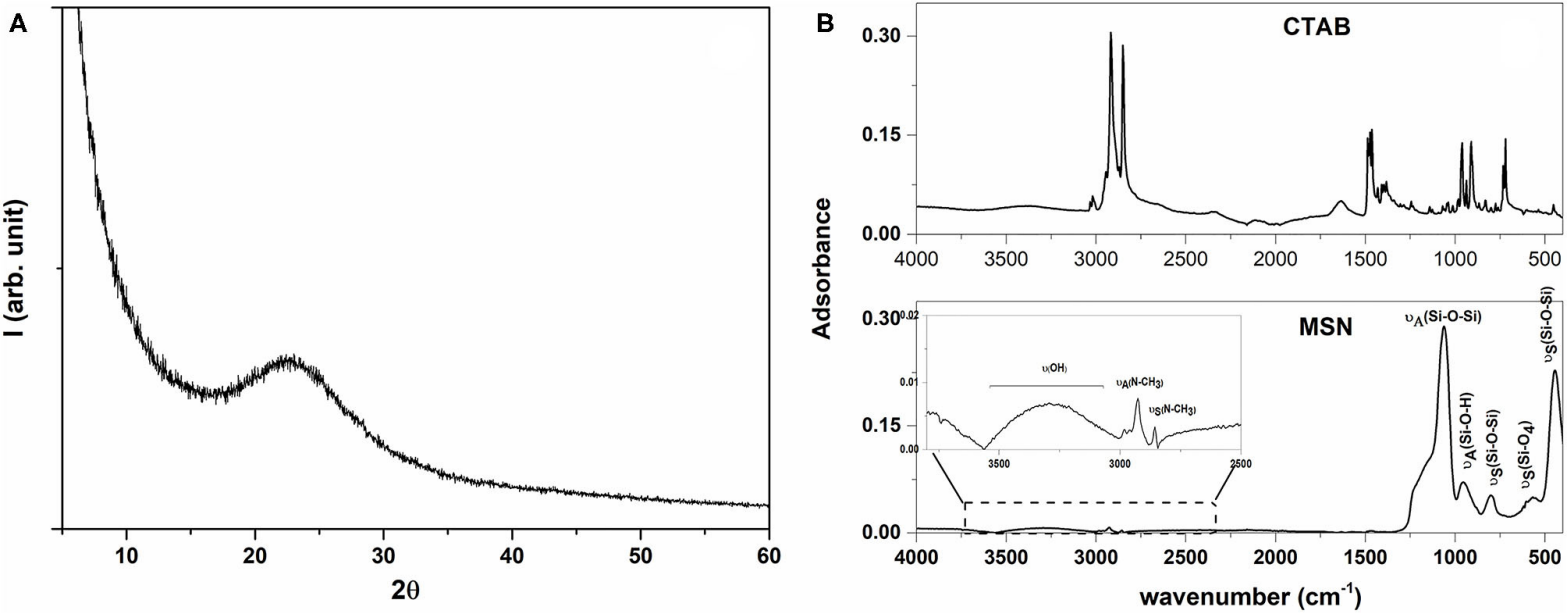
XRD pattern **(A)** of synthesized MSN and FT-IR spectra **(B)** of CTAB and of synthesized MSN.

**Figure 2:**
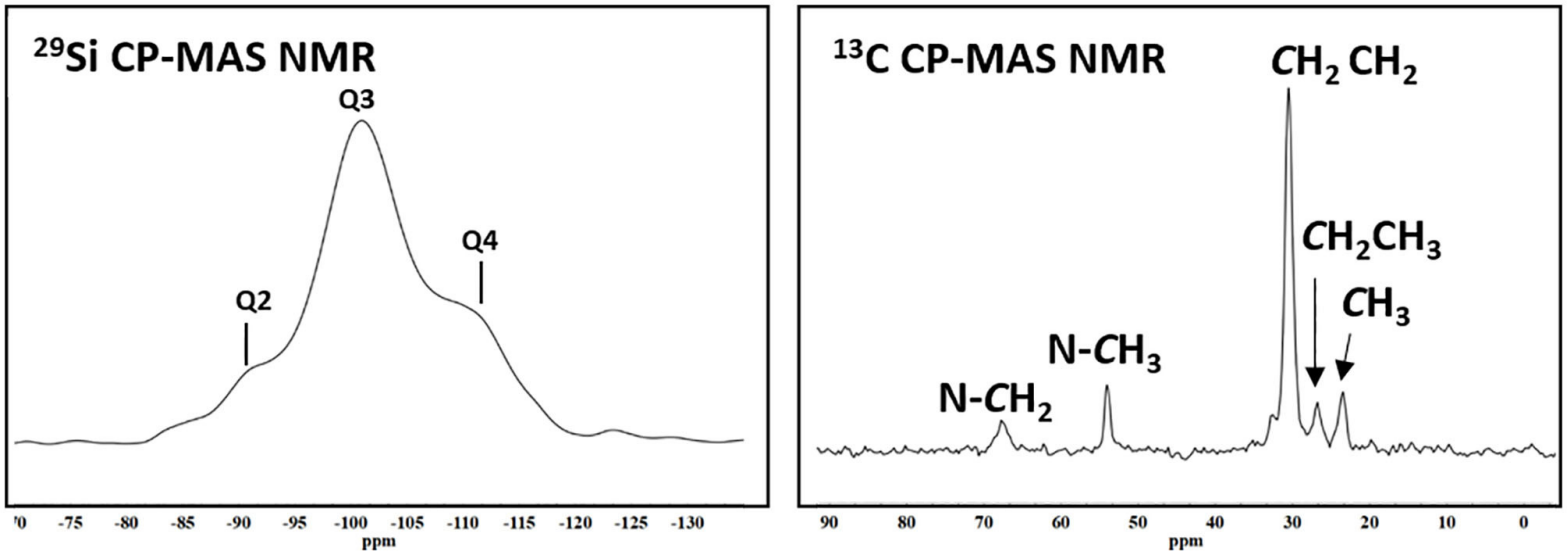
^29^Si CPMAS NMR and ^13^C CPMAS NMR spectra of synthesized MSN.

The physicochemical characterization of the synthesized MSNs was performed to ascertain the goodness of the material used as a carrier in this study. MSNs showed a broad band centered at 22° in the XRD pattern (Figure 1A), which is ascribable to a silica amorphous material (Dubey et al., 2015; Jiang et al., 2019). The ATR spectrum (Figure 1B) of the sample did exhibit the trademark signs of silica (Mourhly et al., 2015). The most intense absorption bands, in the range between 1,000 and 1,300 cm^−1^ are due to the asymmetrical stretching of the Si-O-Si groups (i.e., 1,060 and 1,232 cm^−1^). The signal at 966 cm^−1^ is due to the Si-O symmetric stretching, as well as the ones at 797 and 451 cm^−1^ (Mourhly et al., 2015). The broad band centered at 3,216 cm^−1^ (highlighted in the inlet of the Figure 1B) is due to the stretching of O-H groups, which are indicative of the presence of hydrogen bonds resultant from the interaction occurring between the silanol groups (Si-OH) and the adsorbed water molecules. Accordingly, the signal observed in the ^29^Si CP-MAS NMR spectrum (Figure 2) is due to the convolution of three peaks. The first peak (i.e., Q2; centered at ca. 90 ppm) was due to the geminal silanols, the second peak (i.e., Q3; centered at 100 ppm) highlighted silicon atoms bearing one hydroxyl group, while Q4 peak (centered at around 109 ppm) is due to each Si atom, which is linked over oxygen atoms with 4 Si neighbors (Saladino et al., 2008), which, overall, are contributions of mesoporous silica materials. Other than silica signals, IR spectra revealed vibration bands attributable to CTAB (Viana et al., 2012), as it is evident by comparing the spectrum of the MSN with that of CTAB (upper part of the Figure 1B). The signals falling between 1,250 and 1,500 cm^−1^ were due to the bending vibrational modes of CH_2_ groups, while those at 2,920 and 2,848 cm^−1^ were due to the asymmetric and symmetric stretching of the methyl and methylene groups. CTAB signals were also present in the ^13^C CPMAS NMR spectrum (Figure 2). The resonance of the N-CH_2_ and N-CH_3_ groups was visible at 68 and 58 ppm respectively, while the strong resonance at 30 ppm derived from the methylene chain. Finally, the signals of the CH_2_ and CH_3_ end groups of the CTAB aliphatic chain were clearly visible at 27 and 23 ppm (Xu et al., 2014). These findings evidenced that the CTAB was still present in the material, although several washing steps of MSNs were performed. Indeed, since the bands of ^13^C nuclei were broad, it is reasonable to assert that the mobility of the molecule was low, likely indicating that the residual CTAB was located within the pores of the mesoporous structure (Xu et al., 2014), therefore explaining the reason why it did not remove during the washing steps.

The isotherms of the sample were of Type IV-isotherm, and the hysteresis-type E of “ink-bottle” shape (Figure S2), according to IUPAC classification (Sing et al., 2008; Kraleva et al., 2011). The specific surface area, pore width, and the total pore volume of the synthesized MSN were 422 ± 8 m^2^/g, 3.7 ± 0.1 nm, and 0.90 ± 0.02 cm^3^/g, respectively. The observed high values of specific surface area are in line with those reported elsewhere (Nandiyanto et al., 2009), being also an indication of the mesoporous nature of the obtained silica, which can be used as a carrier because it should load high amount of biocide.

**Figure 3:**
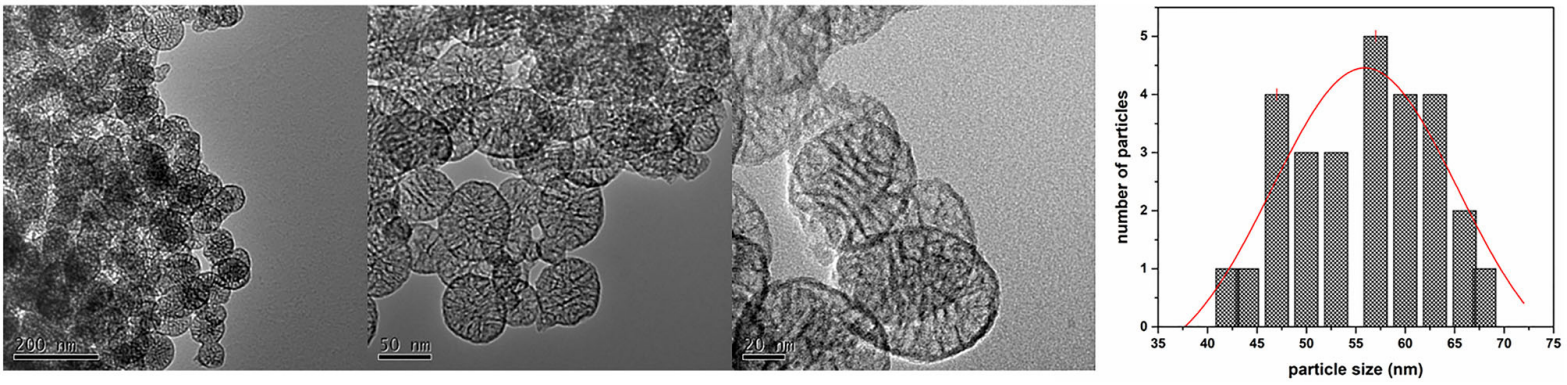
TEM micrographs at different magnification of the obtained MSN and graph of particle size distribution.

Some of the MSN's TEM micrographs at different magnification are reported in Figure 3 together with its size distribution, highlighting how the sample was composed of spherical nanoparticles of 55 ± 10 nm (Figure 3), having also cylindrical pores of ca. three to eight nanometers, which were arranged as parallel arrays concerning the NP diameter. This morphology of MSNs is in good agreement with that of mesoporous nanoparticles investigated elsewhere (Slowing et al., 2008; Cao et al., 2016).

### Maintenance of the Antibacterial Activity of Preventol RI-80 After Loading

**Figure 4:**
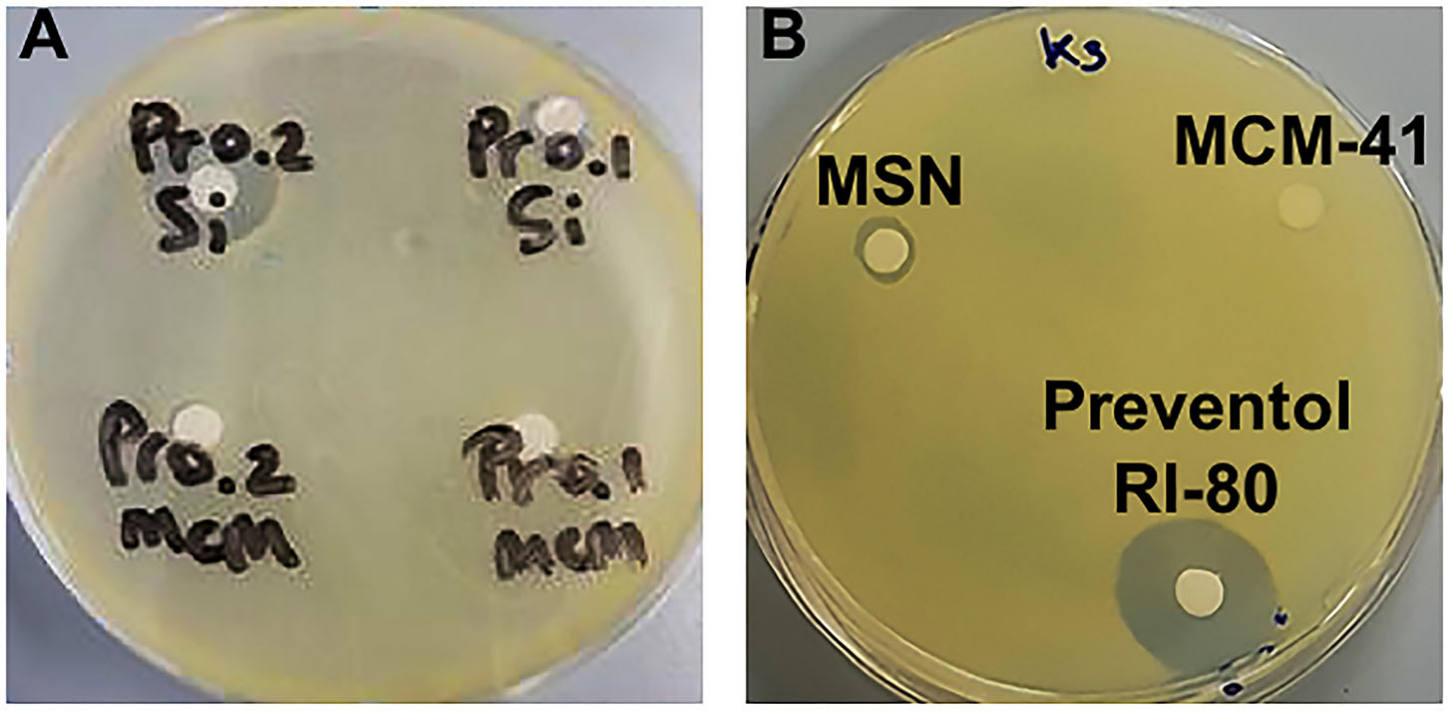
Microbiological assays using *K. rhizophila* as a tester strain. Antibacterial activity **(A)** of Preventol RI-80 0.2@MCM-41 (Pr0.2MCM), Preventol RI-80 0.1@MCM-41 (Pr0.1MCM), Preventol RI-80 0.2@MSN (Pr0.2Si), and Preventol RI-80 0.1@MSN (Pr0.1Si), while in **(B)** is highlighted the effect of either unloaded MSN or MCM-41 systems, as well as Preventol RI-80.

Microbiological assays using decreasing percentages (0.2–0.0002 _v/v_%) of Preventol RI-80 demonstrated that 0.0002 _v/v_% was sufficient to inhibit the growth of *K. rhizophila* (data not shown). Also, the maintenance of the antibacterial activity of the MSN system differently loaded with Preventol RI-80 was compared to that of MCM-41. Microbiological assays showed a large inhibition halo around Preventol RI-80 0.2@MSN than Preventol RI-80 0.1@MSN. Differently, 0.2% of Preventol RI-80 loaded on MCM-41 barely inhibited the bacterial growth, while MCM-41 carrying 0.1% Preventol RI-80 did not exert any antibacterial effect (Figure 4A). This finding demonstrated that the Preventol RI-80 loaded on MSN was more effective than the MCM-41-based system in maintaining the activity against *K. rhizophila*. When we tested the antibacterial activity of 0.2 _v/v_% Preventol RI-80 and either unloaded 0.1% MCM-41 or 0.1% MSN as controls, we found that MCM-41 was completely inactive, while MSN produced a small halo, probably due to the CTAB presence inside the pore structure (Figure 4B). Thus, we surmise that Preventol RI-80@MSN works better than Preventol RI-80@MCM-41 since the antibacterial activity derives from both Preventol RI-80 and, even if at little extent, CTAB.

### Release Studies

**Figure 5:**
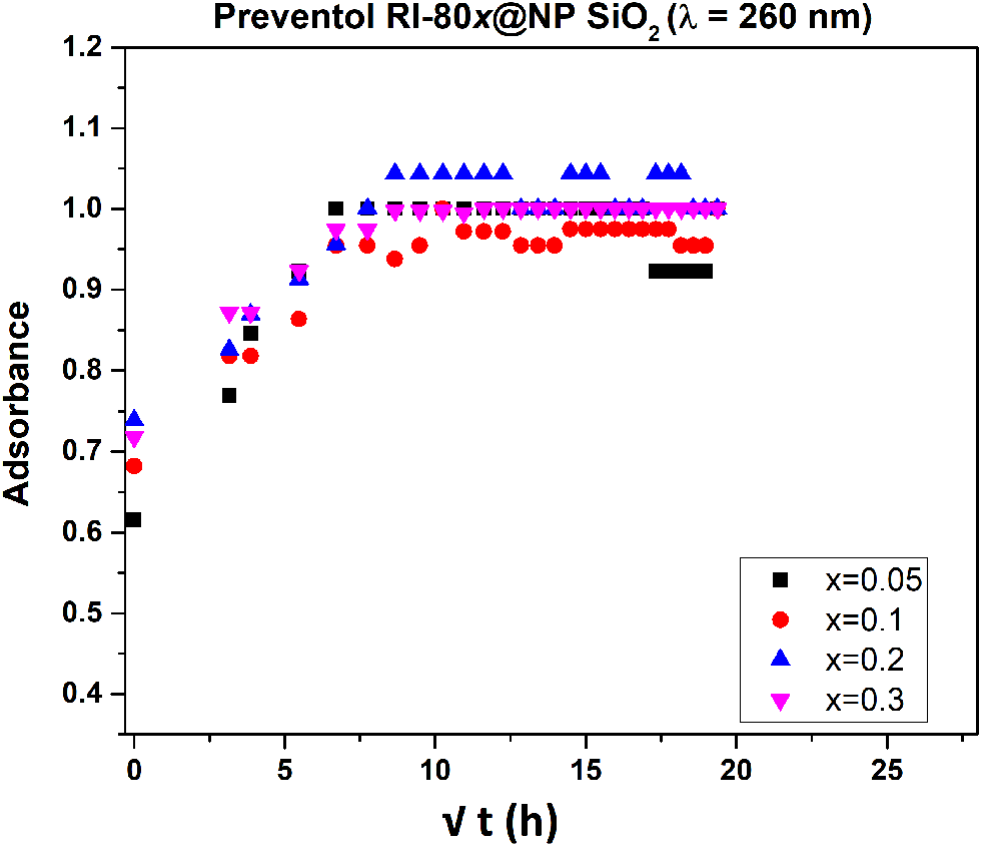
Release profile of Preventol RI-80 from MSN loaded with different amounts of Preventol RI-80 (λ = 260 nm).

To evaluate biocide's release, MSNs loaded with various amounts of Preventol RI-80 were placed in the water and aliquots of the solution were collected at different sampling times. The release kinetics of Preventol RI-80 (Figure 5, Figure S3A) into the water solution was followed by recording intensities at two diverse wavelengths (210 and 260 nm) to determine differences, if any, in the release of the different compounds present within the commercial formulation. Indeed, benzyldimethyltridecylazanium chloride, the main active compound of the quaternary ammonium salts mixture, has the maximum absorbance centered at ca. 260 nm. Preventol RI-80 release was evaluated at the two wavelengths and, although the observed trends were similar, the equilibrium state was reached after either 1 or 3 h for benzyldimethyltridecylazanium chloride or the other components, which have the maximum absorbance at 210 nm, respectively. Accordingly to the literature (Wang, 2009; Bruschi, 2015), the model of Higuchi was applied to the outcomes. The two-step regimes were observed suggesting that the load and then the release is controlled by both chemical and physical entrapping of the active compounds within the pores. The Higuchi constant was in the order Preventol RI-80@MSN (260 nm), *K*_*H*_ = 0.37 ± 0.01 s^−0.5^ > Preventol RI-80@MSN (210 nm), *K*_*H*_ = 0.25 ± 0.01 s^−0.5^, while differences in terms of the amount of biocide release were not observed. Similar results were observed in our previous studies regarding the loading of Biotin-T (constituted by the quaternary ammonium salt didecyldimethylammonium chloride and 2-octyl-2H-isothiazole) (Dresler et al., 2017). This behavior can be explained by a different physicochemical interaction between the hydroxyl groups of the silica and the biocide components and, accordingly to previous study (Dresler et al., 2017), could be ascribed to ion (charged head groups of quaternary salts)—dipole (Si-O-H surface groups) interactions strengthened by the Lewis acid-base interaction among the phenyl group of benzyldimethyltridecylazanium chloride and the acidic sites (electropositive Si) in the MSN structure (Chauhan et al., 2020).

The release efficiency of Preventol RI-80-loaded within MSN was compared with Preventol RI-80 loaded within MCM-41. It is worth mentioning that also MCM41 was efficiently loaded (100%) with Preventol RI-80 as confirmed by the decrease of the specific surface area up to 50 m^2^/g. The release kinetics of Preventol RI-80@MCM-41 followed the Higuchi model (Figures S3B,C); however, the Higuchi constant was in the opposite order as compared to MSNs (i.e., Preventol RI-80@MCM-41 (210 nm), *K*_*H*_ = 0.43 ± 0.02 s^−0.5^ > Preventol RI-80@MCM-41 (260 nm), *K*_*H*_ = 0.26 ± 0.03 s^−0.5^ and, regardless the wavelength, the state of equilibrium was reached roughly after 1 h.

These outcomes suggest a higher efficiency in releasing benzyldimethyltridecylazanium chloride by the Preventol RI-80@MSN as compared with Preventol RI-80@MCM-41 probably due to a higher amount of acidic sites on the surface of MSN thus suggesting that the role of Lewis acid-base interaction is pivotal in regulating the release of biocides. On this basis, the MSN system was judged to be more suitable, in terms of release kinetics, for its application in a real case study; therefore, the biological activity of the Preventol RI-80 0.3@MSN system was compared to that of Preventol RI-80 0.3@MCM-41.

**Figure 6:**
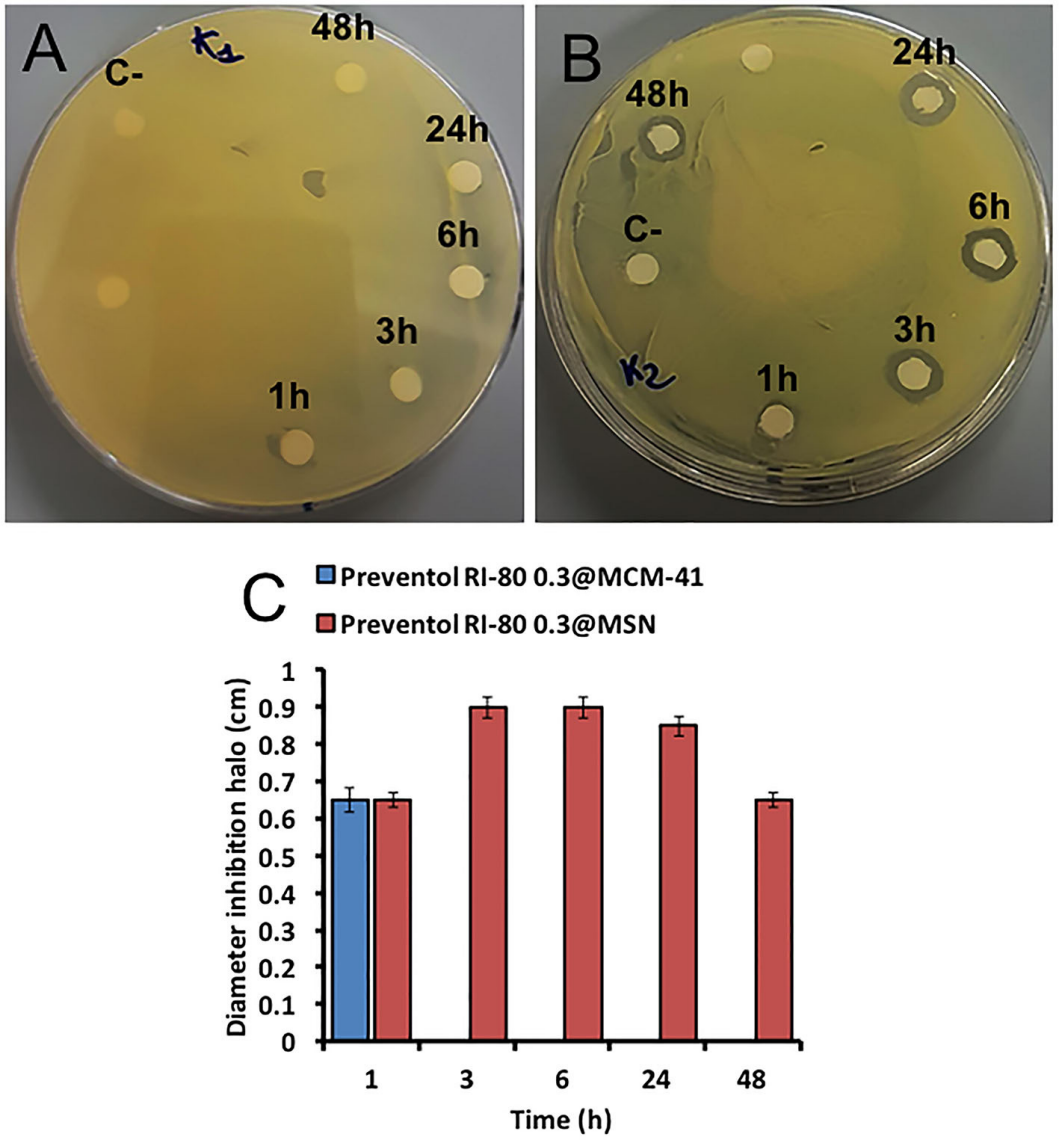
Microbiological assays using *K. rhizophila* as tester strain. Antimicrobial activity of both Preventol RI-80 0.3@MCM-41 **(A)** and Preventol RI-80 0.3@MSN **(B)** CRSs, while the bar graph in **(C)** shows the actual diameter size of the inhibition halos, over time, ascribed to Preventol RI-80 0.3@MCM-41 (blue) and Preventol RI-80 0.3@MSN (red) systems activity.

The biocide release was also studied by microbiological assays for the Preventol RI-80 0.3@MSN system over time (i.e., 1, 3, 6, 24, 48 h), being Preventol RI-80 0.3@MCM-41 one used as a comparison. The system Preventol RI-80 0.3@MSN maintained the antibacterial activity for a longer time than that based on MCM-41; indeed, the latter lost its activity after 1 h of application (Figure 6A), while the system Preventol RI-80 0.3@MSN resulted to be active up to 48 h (Figure 6B). Thus, the MSN-based system resulted to be more suitable as a carrier to achieve both biocide loading and release over time as compared to MCM-41 one, as also indicated by the inhibition halo values (Figure 6C). The difference could be due to CTAB traces in the system, as well as the different size and organization of the mesoporous pore structure. The release of 0.3% Preventol RI-80 was completed within 1 h when MCM-41 was used, while we had previously obtained the complete release of 0.75% Biotin-T from MCM-41 within 6 h (Dresler et al., 2017). Besides the diverse concentrations of biocides used, the difference could be ascribed to the different mechanism of interactions of benzyldimethyltridecylazanium chloride (Preventol RI-80) or 2-octyl-2H-isothiazole (Biotin T) and silica, even it could be influenced by the relative composition of the product (unknown). Therefore, Preventol RI-80 loaded within MSN was the most efficient CRS we designed so far. The release efficiency was in line to that observed for similar silica based CRS developed for the preventive conservation of artifacts (Sorensen et al., 2010; Borisova et al., 2011). On the other hand, it is well-known that the release could be triggered by external stimuli by proper functionalization of silica surface or that the use of layered double hydroxides (LDH) could give a dual role also acting in the capture of specific ions (Giuliani et al., 2020). It has to keep in mind that for applicative purposes, the balance among costs and benefits have to be accounted and, in some cases, low cost materials as natural clays (Cavallaro et al., 2018) could be advantageous.

### Test on Real Case Study: The Stone of the Castellammare Del Golfo's Cave

The Santa Margherita's cave in Castellammare del Golfo (Trapani, Italy) is a natural cave, containing the remains of paintings belonging to an ancient church dated back to the middle age (Purpura, 1999)^1^. The cave is in a poor state of conservation and most of the paintings and the stone are contaminated by biodeteriogens. The identification of biodeteriogenic bacterial strains has been performed (data not shown), as well as the monitoring of environmental conditions (i.e., temperature and humidity), which demonstrated that in a full year there are the proper conditions allowing microbial growth. Thus, fragments of the stone support represent a good case study to test the performances of the MSN system developed to treat damaged stone materials.

**Figure 7:**
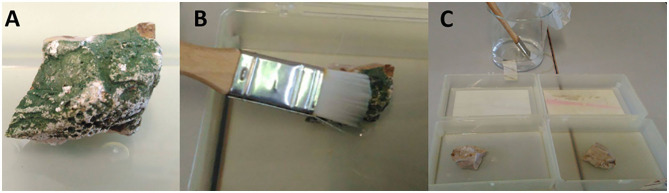
Sample from the Santa Margherita's cave **(A)**, treatment with Preventol RI-80 at 10_v/v_%. **(B)**, and application of the CRS **(C)**.

The Preventol RI-80 0.3@MSN system was applied to a sample taken from the west wall of the cave. The surface of the sample was covered by a green biopatina (Figure 7A), likely representing a microbial biofilm, as microorganisms growing in a sessile life form are more prone in handling harsh environmental conditions and stressors of various nature (i.e., antimicrobials) deriving from the surrounding ecological niche (Piacenza et al., 2017). To simulate a real treatment during the restoration works, the sample was treated with a solution of Preventol RI-80 (10 _v/v_%) for 15 h, being then washed with a water brush (Figure 7B). The green color disappeared after this application. The sample was then divided into two smaller fragments and one of them was used for applying the MSN-based CRS by using a brush (Figure 7C).

The presence of bacteria was evaluated by counting the viable cells and by extracting metagenomics DNA from the untreated stone, the sample treated with Preventol RI-80, and that treated with Preventol RI-80 and Preventol RI-80 0.3@MSN. Evaluation of viable bacteria showed that after 12 months no bacteria were present in samples treated with Preventol RI-80 and additionally with Preventol RI-80 0.3@MSN; differently, from the untreated stone, a mean of 15 ± 2 CFU/g was found at the end of the timeframe considered, while Preventol RI-80 treated samples highlighted the presence of lower microbial contamination after 12 months (Table 1).

**Table 1:** Evaluation of the alive microbial biomass retrieved from either untreated or differently treated stone material.

	**Days of treatment**			
	**2 (days)**	**10 (days)**	**6 (months)**	**12 (months)**
**Sample**	**Colony forming units per gram of stone (CFU/g)**			
Untreated stone	5 ± 2	13 ± 3	10 ± 4	15 ± 2
Preventol RI-80 treated stone	0	0	1 ± 1	3 ± 2
Preventol RI-80@MSN-treated stone	0	0	0	0

**Figure 8:**
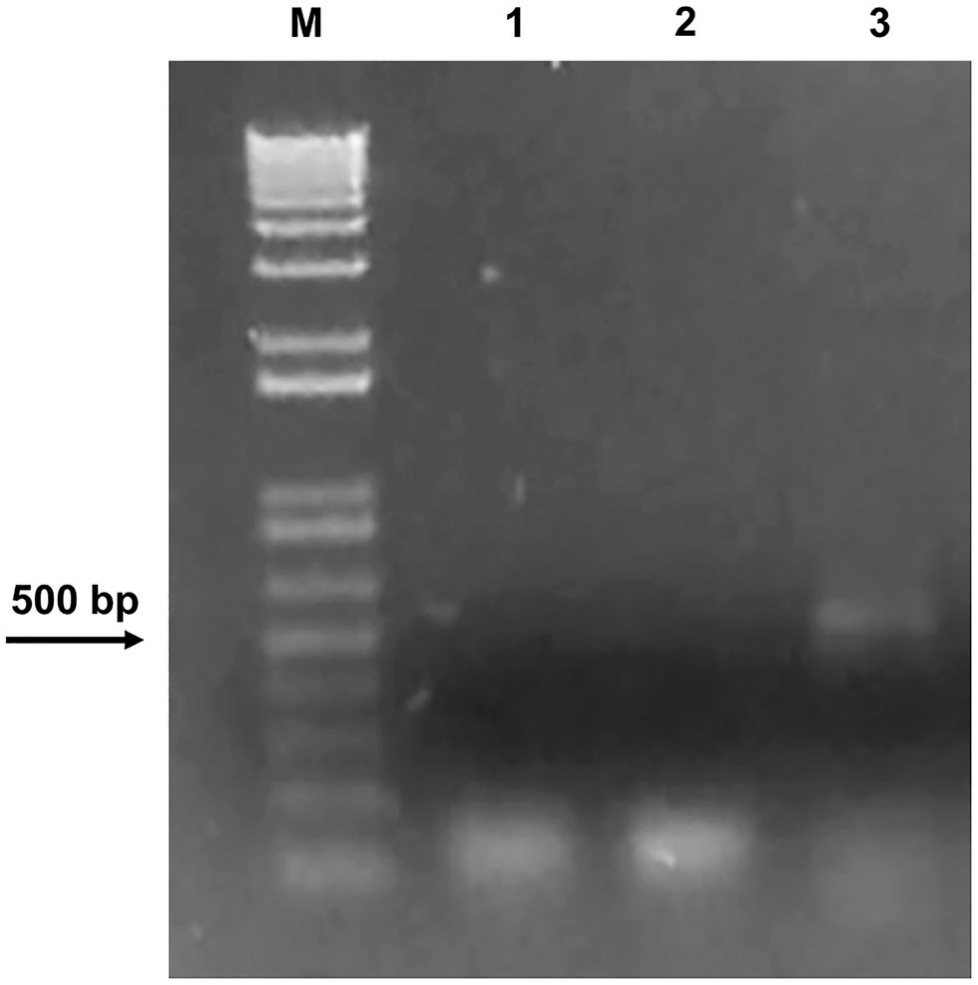
Agarose gel electrophoresis showing DNA only in the untreated stone. M: DNA marker (1 Kb Plus DNA Ladder Biolabs®). PCR was carried out using 1: water as a negative control; 2: DNA extracted from the Preventol RI-80 0.3@MSN-treated stone; 3: DNA extracted from the untreated stone.

We cannot rule out that the initial treatment was so strong to completely inhibit bacterial growth for such a long time or that the conditions in which stones were stored did not allow further bacterial proliferation. Since a minority of environmental microorganisms (ca. 10%) can be isolated through conventional cultivable-dependent methods (Soffritti et al., 2019), to further confirm the absence of bacteria on Preventol RI-80 0.3@MSN-treated stone, metagenomic DNA was extracted from the latter, which was used as a template to amplify a 464-base pair DNA internal fragment of the gene coding for the 16S bacterial ribosomal subunit (Arizza et al., 2019). Agarose gel electrophoresis showed the amplicon of the correct size (~500 bp) only in the untreated stone (Figure 8), thus confirming the efficacy of the treatment.

## Conclusions

This study highlights new avenues in developing controlled release systems directed to a specific target (i.e., stone material) to reduce the frequency of antibacterial treatments of cultural heritage. The mesoporous silica nanoparticles were synthesized by condensation in emulsion method.

The MSNs, spherical in shape featuring having average diameter of 55 nm, show cylinder pores of 3–8 nm in diameter. CRSs based on mesoporous silica nanoparticles with a high yield in loading the biocides Preventol RI-80 were developed and tested as a smart tool against the biodeterioration phenomenon of the material of archaeological and artistic interest.

Preventol RI-80 loaded on MSN showed a high approximation to a more efficient drug delivery system, even higher with respect to the MCM-41.

The antibacterial test showed that all systems are efficient against microbial blooming with MSN more active than MCM-41.

Finally, Preventol RI-80 0.3@MSN system was applied on a stone sample from the Santa Margherita cave in Castellammare del Golfo (Trapani, Italy). The bacterial growth was inhibited for 12 months after treatment. This work demonstrates that it is possible to use the CRSs against the deterioration and that, knowing the kind of biodeteriogens, it is possible to develop *ad hoc* systems to prevent the degradation of artifacts based on stone material. The use of the systems could be extended to other kinds of artifacts based on paper, woods, or metals.

## Data Availability Statement

The raw data supporting the conclusions of this article will be made available by the authors, without undue reservation.

## Author Contributions

RA and MS: Conceptualization and methodology. FA: preparation of samples. RA and AP: biological test. MS: XRD and FT-IR data. AS: NMR data. RA, DC, MS, and AP: writing–review and editing. All authors contributed to the article and approved the submitted version.

## Conflict of Interest

The authors declare that the research was conducted in the absence of any commercial or financial relationships that could be construed as a potential conflict of interest.

## Footnotes

^1^Trapani oggi (2019). Gli affreschi della grotta di Santa Margherita. https://www.trapanioggi.it/gli-affreschi-della-grotta-di-santa-margherita (accessed April 25, 2020).
