# Supplementary material for: Formulation of Mesoporous Silica Nanoparticles for Controlled Release of Antimicrobials for Stone Preventive Conservation
Source: Front Chem. 2020 Aug 21;8:699. doi: 10.3389/fchem.2020.00699 (PMC7471835; doi:10.3389/fchem.2020.00699)
Supplement: Supplementary file 1 [file Table_1.DOCX]

**Support Information**

Formulation of mesoporous silica nanoparticles for controlled release of antimicrobials for stone preventive conservation

Alessandro Presentato^1^, Francesco Armetta^1^, Alberto Spinella^2^, Delia Francesca Chillura Martino^1^, Rosa Alduina^1,*^, Maria Luisa Saladino^1,*^

^1^Department of Biological, Chemical, and Pharmaceutical Sciences and Technology (STEBICEF), University of Palermo, Palermo (Italy)

^2^Advanced Technologies Network (ATeN) Center, University of Palermo, Palermo (Italy)

*

*

*Figure S1. UV Vis Spectrum of Preventol RI-80.*

*Figure S2.* Adsorption-desorption isotherms of *NP SiO_2_, Preventol RI-80 0.3* *v/v%@NP SiO_2_ and Preventol RI-80 0.3* *v/v%@MCM-41.*

*
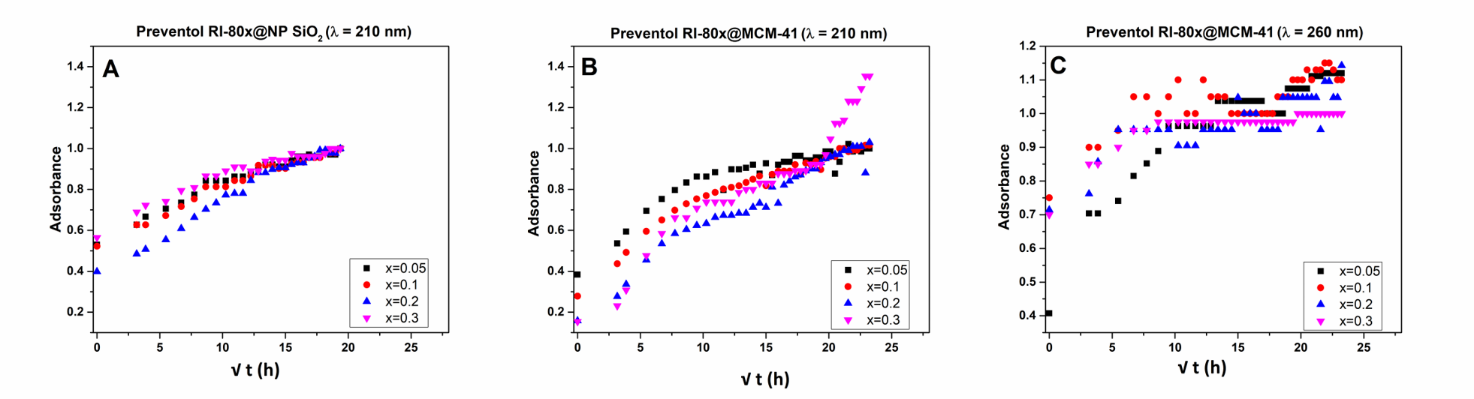
Figure S3.* Release profiles of Preventol RI-80 from MSN and MCM-41 loaded with different amounts of Preventol RI-80 (λ = 210 and 260 nm).
